# Correlation of vascular smooth muscle cell phenotype and cGMP signalling

**DOI:** 10.1186/2050-6511-16-S1-A63

**Published:** 2015-09-02

**Authors:** Moritz Lehners, Hyazinth Dobrowinski, Michael Krämer, Daniel Stehle, Martin Thunemann, Robert Feil

**Affiliations:** 1 Interfakultäres Institut für Biochemie, University of Tübingen, Tübingen, Germany

## 

The phenotypic modulation of vascular smooth muscle cells (VSMCs) is important during vascular development and disease states, such as restenosis and atherosclerosis. It has been suggested that cyclic guanosine monophosphate (cGMP) signalling might be involved in the dynamic changes of VSMC growth and phenotype. Cyclic GMP can be produced by various guanylyl cyclases (GCs). Soluble GC is activated by nitric oxide (NO), whereas the plasma membrane-associated GCs, GC-A and GC-B are stimulated by atrial natriuretic peptide (ANP) and C-type natriuretic peptide (CNP), respectively. To elucidate the potential link between VSMC phenotype and cGMP signalling we isolated and cultured VSMCs from aortae of transgenic mice [R26-CAG-cGi500(L1)] expressing the fluorescence resonance energy transfer (FRET)-based cGMP sensor cGi500 ubiquitously. These cultures underwent different treatments, such as passaging or serum-starvation, to modulate the phenotype of VSMCs. Then the VSMCs were exposed to ANP, CNP, or the NO-releasing compound DEA/NO and intracellular cGMP transients were monitored in real-time at the single-cell level by FRET microscopy. In subsequent steps, the expression of marker proteins for VSMCs was assessed by immunofluorescence staining and correlated with the respective cGMP response of each individual cell. Interestingly, VSMC populations showed heterogeneous cGMP responses with respect to cGMP signals elicited by ANP compared to CNP and these differential responses correlated well with the expression of smooth muscle cell specific marker proteins. VSMCs with a stronger ANP response in comparison to CNP expressed high levels of α smooth muscle actin (α-SMA) and transgelin (SM22α), while cells with a stronger CNP response in comparison to ANP expressed low levels of α-SMA and SM22α. Shifting the VSMC phenotype towards a more contractile phenotype by serum-starvation increased the fraction of cells with strong α-SMA expression and preferential ANP responsiveness. In contrast, promoting the synthetic phenotype by passaging shifted the population towards cells with low α-SMA expression and preferential CNP responsiveness. In conclusion, the differential cGMP responses of VSMCs in culture appear to correlate with distinct phenotypes. Cells with strong responses to ANP exhibit features of differentiated/contractile VSMCs, whereas cells with strong responses to CNP exhibit features of dedifferentiated/synthetic VSMCs. In the future, cGMP imaging in normal and pathophysiologically remodelled vessels should elucidate the relevance of our *in vitro* findings for *in vivo* situations.

